# Screening for triazole resistance in clinically significant *Aspergillus* species; report from Pakistan

**DOI:** 10.1186/s13756-020-00731-8

**Published:** 2020-05-11

**Authors:** Safia Moin, Joveria Farooqi, Kauser Jabeen, Sidra Laiq, Afia Zafar

**Affiliations:** Department of Pathology and Laboratory Medicine, Aga Khan University, Karachi, Pakistan

**Keywords:** Aspergillosis, *Aspergullus flavus*, *Aspergullus fumigatus*, *Aspergullus Niger*, *Aspergullus terreus*, Itraconazole, Voriconazole and posaconazole

## Abstract

**Background:**

Burden of aspergillosis is reported to be significant from developing countries including those in South Asia. The estimated burden in Pakistan is also high on the background of tuberculosis and chronic lung diseases. There is concern for management of aspergillosis with the emergence of azole resistant *Aspergillus* species in neighbouring countries in Central and South Asia.

Hence the aim of this study was to screen significant *Aspergillus* species isolates at the Microbiology Section of Aga Khan Clinical Laboratories, Pakistan, for triazole resistance.

**Methods:**

A descriptive cross-sectional study, conducted at the Aga Khan University Laboratories, Karachi, from September 2016–May 2019. One hundred and fourteen, clinically significant *Aspergillus* isolates [*A. fumigatus* (38; 33.3%), *A. flavus* (64; 56.1%), *A. niger* (9; 7.9%) *A. terreus* (3; 2.6%)] were included. The clinical spectrum ranged from invasive aspergillosis (IA) (*n* = 25; 21.9%), chronic pulmonary aspergillosis (CPA) (*n* = 58; 50.9%), allergic bronchopulmonary aspergillosis (ABPA) (*n* = 4; 3.5%), severe asthma with fungal sensitization (SAFS) (n = 4; 3.5%), saprophytic tracheobronchial aspergillosis (*n* = 23; 20.2%). Screening for triazole resistance was performed by antifungal agar screening method. The minimum inhibitory concentration (MIC) of 41 representative isolates were tested and interpreted according to the Clinical and Laboratory Standards Institute broth microdilution method.

**Results:**

All the isolates were triazole-susceptible on agar screening. MICs of three azole antifungals for 41 tested isolates were found to be ≤1 ml/L; all isolates tested were categorized as triazole-susceptible, including 4 isolates from patients previously on triazole therapy for more than 2 weeks. The minimum inhibitory concentration required to inhibit the growth of 90% organisms (MIC_90_) of itraconazole, voriconazole and posaconazole of the representative *Aspergillus* isolates was 1 mg/L, 1 mg/L and 0.5 mg/L, respectively.

**Conclusion:**

Triazole resistance could not be detected amongst clinical *Aspergillus* isolates from the South of Pakistan. However, environmental strains remain to be tested for a holistic assessment of the situation. This study will set precedence for future periodic antifungal resistance surveillance in our region on *Aspergillus* isolates.

## Background

*Aspergillus* species have emerged as an important cause of morbidity and mortality in the immunocompromised patients with a wide spectrum of aspergillosis requiring systemic antifungal therapy [1]. Invasive pulmonary aspergillosis (IPA) is a life threatening pneumonia characterized by lung parenchymal invasion with vascular erosion and necrosis [2]. Furthermore, *Aspergillus* species also produce a wide range of chronic, saprophytic, and allergic conditions. Chronic pulmonary aspergillosis (CPA) can be a debilitating illness, progressing rapidly as in sub acute invasive aspergillosis (SAIA) or more slowly as chronic cavitary pulmonary aspergillosis (CCPA) and chronic fibrosing pulmonary aspergillosis (CFPA). Thus the clinical spectrum of aspergillosis requiring systemic therapy is very wide, ranging from fulminant invasive disease in patients with no immune defenses to slowly progressing fibrosing disease as seen in CFPA and severe asthma with fungal sensitization (SAFS) or allergic bronchopulmonary aspergillosis (ABPA).

Burden of IA and the other forms of aspergillosis have been reported to be significant from developing countries including South Asia and South East Asia [3]. Due to inadequate population based surveillance, the accurate burden of fungal infection in Pakistan has not been documented. However infections prevailing in our country project a high fungal burden and an estimate has been drawn for the major fungal burden with the help of data from neighboring countries like India [4].

*Aspergillus fumigatus* is the predominant etiological agent isolated from IPA cases closely followed by *Aspergillus flavus* in South Asia [5–8]. Long term triazole therapy is recommended for the treatment of IPA [9]. Management of IPA and the other forms of aspergillosis has become challenging with the emergence of azole resistance in *Aspergillus* species especially *Aspergillus fumigatus* [10]. Long-term azole therapy and the unrestricted use of azole compounds in the environment as fungicides in the agricultural and horticultural industry have been identified as risk factors for development of resistance [10]. Some of the fungicides have a similar molecule structure to the triazoles used in treatment and hence lead to azole resistance. This is of great importance to an agricultural country like Pakistan.

Due to the life threatening nature of these infections, surveillance for resistance has become extremely important [1, 11]. Standard susceptibility testing method is broth microdilution for molds according to the European Committee on Antibiotic Susceptibility Testing (EUCAST) and Clinical and Laboratory Standards Institute (CLSI) [1]. This technique is technically challenging and time consuming. Therefore the strategy adopted by most clinical laboratories is to first screen *Aspergillus* species for triazole resistance. The isolates that are initially found to be resistant on screening are further tested by the standard broth microdilution.

Hence the aim of this study was to screen significant *Aspergillus* species isolates at the Microbiology Section of Aga Khan Clinical Laboratories for triazole resistance. This will set precedence for future periodic antifungal resistance surveillance studies in our region on *Aspergillus* isolates causing invasive disease, as well as other syndromes requiring long term antifungal therapy.

## Materials and methods

The aim of this study was to screen significant *Aspergillus* species isolates. It was a prospective, descriptive cross-sectional study conducted in the Aga Khan University Clinical Laboratories, Karachi, from September 2016 to May 2019.

### Identification and susceptibility testing

*Aspergillus* species isolated from the clinical specimen from patients with appropriate host factors and sufficient clinical evidence consistent with the above mentioned clinical spectrum were included in the study. They were assessed for their clinical significance using EORTC 2008, *Asp*ICU, and the clinical spectrum of pulmonary aspergillosis [12–17]. Non *Aspergillus* species were excluded. The *Aspergillus* isolates were identified based on the colony morphology and microscopic morphology [18]. Inoculum was prepared according to CLSI [19]. Briefly, the *Aspergillus* isolates were grown on potato dextrose agar slants for 5 days at 35 °C or until good sporulation was observed. The sporulating colonies were covered with 1 mL of sterile 0.85% saline and the colonies were gently probed with the tip of a transfer pipette to prepare a suspension. The resulting mixture of conidia, sporangiophores and hyphal fragments was withdrawn and transferred to a sterile tube and after allowing the heavy particles to settle for three to 5 min, the upper homogenous suspension was transferred to another sterile tube. The cap was tightened and the suspension vortexed thoroughly for 15 s. The conidia or sporangiophore suspension was adjusted to an optical density (O.D.) at 530 nm that ranges from 0.09 to 0.13 for *Aspergillus* spp.

**Fig. 1:**
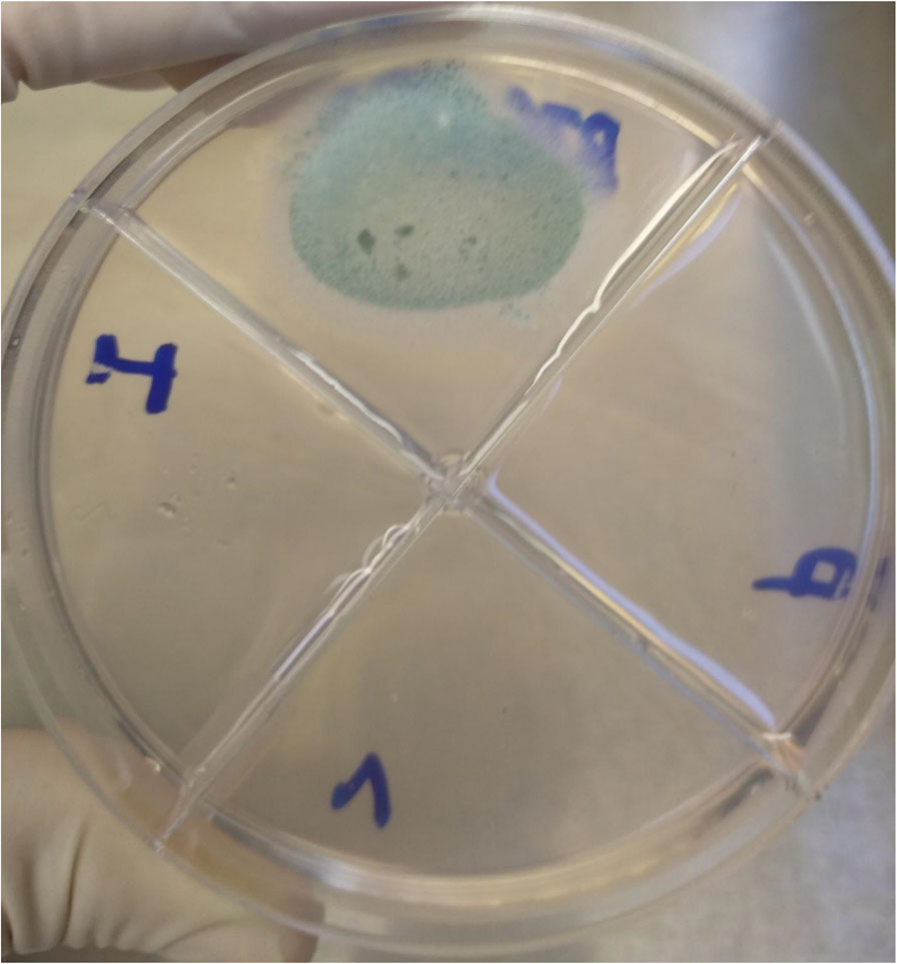
*Aspergillus fumigatus* isolate recovered from an Invasive Pulmonary Aspergillosis patient, found susceptible to the triazoles on agar screening. Itraconazole 4 mg/L in left quadrant, Voriconazole 1 mg/L below and on the right, Posaconazole 0.5 mg/L. Growth control is on the top quadrant

Screening for triazole resistance was performed by antifungal agar screening method as described by Mortensen et al [20] Itraconazole, voriconazole and posaconazole, powders from the Sigma-Aldrich Company (St. Louis, MO, USA) were used to prepare the agar screening plates. Subsequently 50 μl was inoculated in each well of a four-well petri plate containing Roswell Park Memorial Institute (RPMI) 1640 agar with 2% glucose supplemented with itraconazole (4 mg/L), voriconazole (1 mg/L), and posaconazole (0.5 mg/L), and no antifungal (positive-control well) (Fig. 1). Plates were incubated at 35 °C and examined for growth at 24, 48, and 72 h. The interpretation of the plates was as follows. If colonies of an *Aspergillus* spp. were observed in the control well and no growth in azole wells of the four-well petri plate, the isolate was categorized as an azole-susceptible *Aspergillus* spp. If colonies of *Aspergillus* grew on the azole wells, then it was categorized as an azole-non-susceptible *Aspergillus* spp. All the tested isolates were found to be susceptible to the triazoles.

The minimum inhibitory concentration (MIC) of representative isolates {*A. flavus* [*n* = 15(13.2%)], *A. fumigatus* [*n* = 15(13.2%)], *A. niger* [*n* = 8(7.02%)] and *A. terreus* [*n* = 3(2.6%)]} was tested according to the CLSI broth microdilution method [19]. Briefly, 96-well U-bottom micro titer plates (Costar, Corning Incorporated) were used. Drug dilutions were prepared following the 2-fold drug dilution scheme described in document M38-A2 to yield serial 2-fold drug dilutions (from 0.03 to 16 mg/L), which were twice as concentrated as the final concentrations. The microdilution plates were stored at − 70 °C until use. *Aspergillus* conidial inoculum suspensions were prepared as described above to an O.D. of 0.9–0.13. This suspension was then diluted 1:50 times with RPMI 1640 broth to give a final inoculum density of 0.4 × 10^4^-5 × 10^4^ CFU/ml in 200 μL when added to an equal volume of drug suspension in the microtiter plate well, as confirmed by quantitative inoculum controls [19]. Growth (drug-free) and fungus-free controls were included. The microdilution plates were incubated at 35 °C and examined for the MICs after 46 to 50 h. The MIC was the lowest drug concentration that prevented any discernible growth (100% inhibition).

Interpretation of results: Breakpoints for mold testing have not been established by the CLSI. However, working breakpoints have been assigned as follows [19]:

Itraconazole: susceptible (MIC ≤1 mg/L), intermediate (MIC 2 mg/L) and resistant (MIC ≥4 mg/L). Voriconazole: susceptible (MIC ≤1 mg/L), intermediate (MIC 2 mg/L) and resistant (MIC ≥4 mg/L). Posaconazole: susceptible (MIC ≤1 mg/L), intermediate (MIC 2 mg/L) and resistant (MIC ≥4 mg/L).

American Type Culture Collection (ATCC) strains used as controls in the susceptibility testing were *Candida parapsilosis* ATCC 22019, *Candida krusei* ATCC 6258 and *A. flavus* ATCC 204304. The quality control results were within acceptable quality control ranges on all days of testing. Ethical exemption was obtained from the institutional ethics review committee.

#### Sample size

Sample size was calculated using OpenEpi online software for “Sample Size for a Proportion or Descriptive Study” and the formula used was n = [DEFF*Np (1-p)]/ [(d2/Z21-α/2*(N-1) + p*(1-p)]. The number of patients registered with AKUH and diagnosed with aspergillosis (according to ICD-09), in 2015, were 49 [21]. Hence, using this population, one hundred and fourteen non-duplicate *Aspergillus* isolates were sampled to detect a hypothesized frequency of triazole resistance of 1.7 + 1% at 95% level of confidence [8]. Non-probability consecutive sampling was used.

## Results

One hundred and fourteen, clinically significant *Aspergillus* isolates [*A. fumigatus* (38; 33.3%), *A. flavus* (64; 56.1%), *A. niger* (9; 7.9%) and *A. terreus* (3; 2.6%)] were included in the study. The clinical spectrum included invasive aspergillosis (IA) (*n* = 25; 21.9%) further divided into proven invasive extrapulmonary aspergillosis (*n* = 8; 7%), proven invasive pulmonary aspergillosis (IPA) (*n* = 6; 5.3%), putative/probable IPA (*n* = 11; 9.6%). Eighty nine isolates from patients with chronic pulmonary aspergillosis (CPA) (*n* = 58; 50.9%), allergic bronchopulmonary aspergillosis (ABPA) (*n* = 4; 3.5%), severe asthma with fungal sensitization (SAFS) (n = 4; 3.5%) and saprophytic tracheobronchial aspergillosis (*n* = 23; 20.2%) were also included.

We included samples from nine children (younger than 18-yr-old). One had IPA with *A. fumigatus* (Fig. 1), and three suffered from ABPA (*A. flavus, A. fumigatus, A. terreus*) two of whom improved on itraconazole. Three more children had SAFS (*A. flavus, A. fumigatus, A. niger*). One 16-year-old girl had post-tuberculosis CPA with *A. flavus*. One was saprophytic tracheobronchial aspergillosis with *A. fumigatus*. The male to female ratio of our patients was 2.0 (Table 1).

**Table 1:** Age and gender distribution of the *Aspergillus* strains isolated from a clinical spectrum of aspergillosis

	n (%)	Age range (y)	Gender (M:F)
No. of isolates	114	6–90	2.0
Clinical diagnoses			
Extrapulmonary	8 (7.0)	20–60	1.6
IA proven	8 (7.0)	20–60	1.6
Pulmonary	103 (90.4)	6–90	2.0
IPA proven	6 (5.3)	11–60	2.0
IPA putative/probable	11 (9.6)	22–81	1.8
CPA	58 (50.9)	16–90	1.7
ABPA	4 (3.5)	6–32	1.0
SAFS	4 (3.5)	6.5–64	1.0
Saprophytic tracheobronchial aspergillosis	23 (20.2)	15–90	0.2
Previously triazole exposed isolates	5 (4.4)	40–65	0.7

*IA* Invasive Aspergillosis, *IPA* Invasive Pulmonary Aspergillosis, *CPA* Chronic Pulmonary Aspergillosis, *ABPA* Allergic Bronchopulmonary Aspergillosis, *SAFS* Severe Asthma with Fungal Sensitization

All the isolates were categorized as triazole-susceptible based on the triazole antifungal agar screening. The MICs of the three azole antifungals for 41 representative isolates tested were found to be ≤1 mg/L and hence according to CLSI breakpoints, all the isolates tested were found to be triazole-susceptible. The minimum inhibitory concentration required to inhibit the growth of 90% organisms (MIC90) of itraconazole, voriconazole and posaconazole of the representative *Aspergillus* isolates was 1 mg/L, 1 mg/L and 0.5 mg/L respectively (Tables 2 and 3).

**Table 2:** MIC range and MIC90 of triazoles of four commonly isolated *Aspergillus* spp. in clinically significant cases

	Isolates	*Aspergillus flavus*	*Aspergillus fumigatus*	*Aspergillus niger*	*Aspergillus terreus*
Total isolates tested [n (%)]	41 (36)	15 (13.2)	15 (13.2)	8 (7)	3 (2.6)
MIC range itraconazole (mg/L)	0.06–1	0.06–1	0.25–1	0.25–1	0.25
MIC90 itraconazole (mg/L)	1	0.4	0.5	1	0.25
MIC range voriconazole (mg/L)	0.125–1	0.125–1	0.25–1	0.25–0.5	0.5–1
MIC90 voriconazole (mg/L)	1	1	1	0.5	0.9
MIC range posaconazole (mg/L)	0.06–1	0.06–1	0.25–1	0.25–0.5	0.5
MIC90 posaconazole (mg/L)	1	0.5	0.5	0.5	0.5

*MIC* Minimum Inhibitory Concentration, *MIC90* Minimum Inhibitory Concentration to inhibit the growth of 90% organisms

**Table 3:** MIC range and MIC90 of triazoles of *Aspergillus* spp. according to the clinical spectrum

	Isolates	*Aspergillus flavus*	*Aspergillus fumigatus*	*Aspergillus. niger*	*Aspergillus terreus*
Total isolates screened [n (%)]	114	64 (56.1)	38 (33.3)	9 (7.9)	3 (2.6)
Extrapulmonary IA proven [n (%)]	8 (7.0)	7 (6.1)	0	0	1 (0.9)
MIC tested [n (%)]	7 (6.1)	6 (5.3)	–	–	1 (0.9)
MIC Itraconazole range (mg/L)	0.25–0.5	0.25–0.5	–	–	0.25
MIC Voriconazole range (mg/L)	0.5–1	0.5–1	–	–	1
MIC Posaconazole range (mg/L)	0.06–0.5	0.06–0.5	–	–	0.5
Invasive Pulmonary Aspergillosis (Proven, Putative/Probable)	17 (14.9)	12 (10.5)	4 (3.5)	1 (0.9)	0
MIC tested [n (%)]	9 (7.9)	4 (3.5)	4 (3.5)	1 (0.9)	0
MIC Itraconazole range (mg/L)	0.06–0.5	0.06–0.25	0.25–0.5	0.5	0
MIC Voriconazole range (mg/L)	0.5–1	0.5–1	0.5–1	0.5	0
MIC Posaconazole range (mg/L)	0.5	0.5	0.5	0.5	0
Non-Invasive Pulmonary Aspergillosis (CPA, ABPA, SAFS)	66 (57.9)	37 (32.5)	21 (18.4)	6 (5.3)	2 (1.8)
MIC tested [n (%)]	22 (19.3)	5 (4.4)	10 (8.8)	5 (4.4)	2 (1.8)
MIC Itraconazole range (mg/L)	0.25–1	0.25–1	0.25–1	0.25–1	0.25
MIC Voriconazole range (mg/L)	0.125–1	0.125–1	0.25–1	0.25–0.5	0.5
MIC Posaconazole range (mg/L)	0.25–1	0.5–1	0.25–1	0.5	0.5
Saprophytic tracheobronchial Aspergillosis [n (%)]	23 (20.2)	8 (7.0)	13 (11.4)	2 (1.8)	0
MIC tested [n (%)]	3 (2.6)	–	1 (0.9)	2 (1.8)	–
MIC Itraconazole range (mg/L)	0.25–1	–	0.25	0.5–1	–
MIC Voriconazole range (mg/L)	0.25–1	–	1	0.25–0.5	–
MIC Posaconazole range (mg/L)	0.25–0.5	–	0.5	0.25–0.5	–

*IA* Invasive Aspergillosis, *CPA* Chronic Pulmonary Aspergillosis, *ABPA* Allergic Bronchopulmonary Aspergillosis, *SAFS* Severe Asthma with Fungal Sensitization, *MIC* Minimum Inhibitory Concentration

Extrapulmonary IA (Table 3) cases included three *A. flavus* brain abscesses, two of whom were treated with voriconazole. One of these patients expired, while the other had an exposure of 28 days to voriconazole before this isolate was recovered, and was again discharged from the hospital on it. All three of these isolates had voriconazole MIC of 1 mg/L while itraconazole and posaconazole MICs were < 0.5 mg/L. We also had an *A. terreus* mitral valve endocarditis case (Table 3), with a positive galactomannan assay and beta d-glucan assay and had been started empirically on fluconazole. Unfortunately, the patient was not admitted at our hospital and was lost to follow-up after the report was finalized. We also tested three *A. flavus* strains causing invasive sinusitis (Table 3), one of which was previously exposed to voriconazole.

Of the 17 IPA cases for which we also determined antifungal MICs (Table 3), four patients suffered from hematological or solid organ malignancy. Voriconazole was the most common choice of antifungal in both the oncology and pulmonology patients, with the MIC_90_ of 0.95 mg/L for all *Aspergillus* species. There were IA in critically ill ICU patients (*n* = 5, all *A. flavus*) with two of them having positive galactomannan and beta d-glucan assay. One patient received voriconazole which was a good treatment option with an MIC of 0.5 mg/L.

Our study included 58 isolates from patients with CPA (CNPA *n* = 10, CCPA *n* = 6, CFPA *n* = 13, aspergilloma n = 5). One patient with the clinical spectrum bordering on CCPA/CFPA and a recurrent infection with *A. niger*, had received prolonged treatment with itraconazole for 197 days. Two of the CPA patients were treated with voriconazole (MIC of both was 0.5 mg/L) while another two received itraconazole, with one having prolonged exposure; itraconazole MICs of both these isolates was 0.25 mg/L. Two of our ABPA patients were discharged on itraconazole, MIC of both the isolates was 0.25 mg/L.

Five of our isolates were previously exposed to triazoles, as mentioned above. Four of them were *A. flavus*, out of which two were proven IA; one was a brain abscess, who had an exposure of 26 days to voriconazole (MIC voriconazole was 1 mg/L) and was discharged on it. The other *A. flavus* IA was isolated from a case of recurrent sinusitis, who had an exposure of 47 days to voriconazole (MIC voriconazole was 1 mg/L) and was discharged on it. The third one was a probable IPA in a neutropenic patient of relapsed acute myelogenous leukemia who had an exposure of 17 days to voriconazole (MIC voriconazole was 1 mg/L). Unfortunately the patient expired, unclear whether it was due to primary disease or the fungal infection. The fourth one was a CPA patient who was exposed to itraconazole (MIC itraconazole was 0.25 mg/L). This was an outpatient and was lost to follow up and the duration of exposure could not be ascertained. The fifth was a patient with the clinical spectrum bordering on CCPA/CFPA and a recurrent infection with *A.niger*, who had received prolonged treatment with itraconazole for 197 days when the sample was submitted, and was still under treatment with it. (MIC itraconazole was 0.25 mg/L).

## Discussion

In the present study, we examined itraconazole, voriconazole and posaconazole susceptibility in a spectrum of clinically significant *Aspergillus* spp. *A. flavus* was found to be the most common clinically significant *Aspergillus* spp. in this study followed by *A. fumigatus, A. niger* and *A. terreus*, respectively. We screened 114 isolates for azole resistance but we could not detect any triazole resistant *Aspergillus* isolate. MICs of representative isolates were tested by broth microdilution and all of them were found to be triazole susceptible. Hence, triazole resistance could not be detected in clinical *Aspergillus* isolates from a single laboratory in our country over the last 4 years. Our data can nevertheless serve as baseline for future surveillance of triazole susceptibility in clinically significant *Aspergillus* spp. in our region, as such data in either clinical or environmental *Aspergillus* isolates is currently lacking from Pakistan.

Resistant isolates harboring either TR34/L98H or TR46/Y121F/T289A mutations have been found in environmental and clinical samples from several countries including the USA, United Kingdom, Ireland, most countries in Europe, Tanzania, and Australia [22]. Arendrup et al. screened a total of 3788 *Aspergillus* isolates for azole resistance from a similar clinical spectrum to our study, but as a multicenter international surveillance during January 2009–January 2011. *A. fumigatus* species complex constituted 77.6% of their isolates, while in our study, *A. flavus* was the most common species (56.1%), and *A. fumigatus* was only 33.3% of the isolates. Acquired azole resistance in *A. fumigatus* was detected in 11 of 17 European centers in nine countries, with an overall 3.2% prevalence of azole resistance. The median MICs of itraconazole, voriconazole and posaconazole for the resistant isolates were > 8, 2 and 1 mg/L respectively. TR34/L98H was the predominant mechanism of resistance. Our isolates showed lower MICs. The MIC90 of itraconazole, voriconazole and posaconazole of our *A .fumigatus* isolates were 0.5, 1 and 0.5 mg/L respectively (Table 2) [23, 24].

Data on azole resistant *Aspergillus* spp. from clinical and environmental isolates is also available from Far East: Taiwan, Thailand, Japan; and our neighboring countries, India, China, and Iran. Tashiro et al. confirmed that long-term itraconazole therapy induced azole resistance in *A. fumigatus* isolates with itraconazole MICs ≥4 mg/L is isolates that were exposed to itraconazole for > 115 days [24]. Five of our isolates were previously exposed to azoles, as described above. However, we could not detect any triazole resistance in our isolates. One of these had an exposure of 197 days to itraconazole, but its MIC was 0.25 mg/L. Liu et al., from China identified four azole resistant *A. fumigatus* strains out of 72 clinical isolates, based on mutations of cyp51A. Three strains were highly resistant to itraconazole (MIC 16 mg/L), two of which exhibited the TR34/L98H/S297T/F495I mutation, while one carried only the TR34/L98H mutation. The fourth multiazaole-resistant isolate (itaconazole 4 mg/L, voriconazole 2 mg/L) carried a new G432A mutation [25]. Chowdhary et al. found azole resistance in [12] 1.7% *A. fumigatus* clinical isolates during 4 years in a referral Chest Hospital in Delhi, India. These isolates harbored TR_34_/L98H mutation in 83.3% isolates with a pan-azole resistant phenotype, linked to the use of fungicide azoles in agricultural practices. Of the 12 resistant *A. fumigatus*, 11 showed a pan-azole resistant phenotype exhibiting high MIC of all the triazoles, itraconazole [geometric mean (GM) MIC = 16 mg/L], voriconazole (GM MIC = 8 mg/L), and posaconazole (GM MIC = 2.82 mg/L). In contrast, a solitary *A. fumigatus* isolate exhibited high MIC (> 16 mg/L) against itraconazole only [8]. Chowdhary et al. also reported 7% triazole resistance in *A. fumigatus* isolates from 24 environmental samples in India, which shared the same TR34/L98H mutation in the cyp51 gene and showed cross-resistance to itraconazole, voriconazole and posaconazole, and to six triazole fungicides used extensively in agriculture. The mutated environmental strains showed high MICs (itraconazole GM MIC = 16 mg/L], voriconazole (GM MIC = 8.7 mg/L), and posaconazole (GM MIC = 1.03 mg/L), and the mutated clinical isolates showed the following MICs (itraconazole GM MIC = 16 mg/L], voriconazole (GM MIC = 5.9 mg/L), and posaconazole (GM MIC = 3.2 mg/L) [26] [26]. Nabili et al. in a three-year study, screened 513 samples (213 clinical and 300 environmental samples) from ten provinces of Iran for azole resistance, and found a 6.6% prevalence of azole-resistant *A. fumigatus* in Iran ([clinical and environmental *A. fumigatus* isolates with decreased drug susceptibility and mutations: itraconazole MIC range 4 to > 16 mg/L, voriconazole MIC range 0.25 to > 16 mg /L posaconazole MIC range 0.016 to 4 mg/L]. Among resistant isolates, TR34/L98H mutations in the CYP51A gene were the most prevalent (80%) [27]. In comparison to these studies, our isolates showed comparatively lower MICs.

J.F. Meis and, A. Chowdhary et al. recently sequenced and analysed 24 genomes of *A. fumigatus* from across the world. These isolates were rationally chosen to include an informative selection of clinical and environmental isolates that were wild-type, or known to carry the TR34/L98H allele. This population genomic analysis showed that *A. fumigatus* was broadly panmictic, with as much genetic diversity found within a country as is found between continents [28].

The occurrence of azoles resistant isolates of *A. fumigatus* varies worldwide, from 2.1–20% in the UK, 10–12% in the Europe, 10% in Asia, Africa, America and Australia to 1.75% in India, probably due to varying usage of azole fungicides that may select for resistance [29].

Amongst our isolates*, A. flavus* was the most common one. At present, the development of azole resistance is mainly associated with *A. fumigatus* and less so with *A. flavus* and *A. terreus*; however, further surveillance is warranted. *A. flavus* is the cause of a broad spectrum of human diseases predominantly in Asia, the Middle East, and Africa possibly due to its ability to survive better in hot and arid climatic conditions compared to other *Aspergillus* spp. [30]. In a collection of 590 clinical isolates, from five centers in USA and Europe, the rate of voriconazole resistance in *A. flavus* was estimated at ~ 2% using an ECV of > 1 μg/mL [31].

There is limited data from Pakistan on the mold antifungal surveillance. A study was conducted in the Armed Forces Institute of Pathology, Rawalpindi from January through December, 2016. 110 isolates; 45 (40.9%) *A. fumigatus*, 40 (36.3%) *Alternaria alternata*, and 25 (22.7%) *Cladosporium sphaerospermum* were tested against amphotericin B, fluconazole and voriconazole by broth microdilution method. The overall voriconazole and amphotericin B susceptibility rates were 82.2 and 84.5%, respectively. Voriconazole resistance was seen in only 1 (2.5%) *A. fumigatus* isolate, which was not confirmed by genotyping [32]. Due to our limited resources, our study also lacks the detection of molecular mechanisms of azole resistance in our clinical isolates.

The limitations of our study are the small sample size, and single centre which couldn’t find any triazole resistant *Aspergillus* isolate. However, we feel the bench experience will help introduce the screening and MICs into routine clinical practice, though Azole resistant *Aspergillus* disease is a rare entity and difficult to diagnose [10]. Unfortunately, the susceptibilities could not be performed in real-time but in batches, due to logistic issues which reduced the clinical utility for some patients.

Future directions necessitate surveillance of our environmental and clinical isolates, to determine any rising MICs, and change in our local epidemiology. This MIC data should be linked to clinical antifungal usage and therapeutic drug monitoring in patients who are on long-term azole therapy, as in CPA cases. Another concern is the missing data on the quantity and the types of antifungals used in agriculture, as Pakistan is an agricultural economy and there is high risk of excessive use.

## Conclusion

Triazole resistance could not be detected amongst clinical *Aspergillus* isolates from the South of Pakistan. However, environmental strains remain to be tested for a holistic assessment of the situation. Thus this study sets precedence for future periodic antifungal resistance surveillance studies in our region on *Aspergillus* isolates causing invasive disease, as well as other syndromes requiring long term antifungal therapy.

## Data Availability

The datasets used and/or analysed during the current study are available from the corresponding author on reasonable request.

## Abbreviations

IPA: Invasive Pulmonary Aspergillosis
CPA: Chronic Pulmonary Aspergillosis
SAIA: Sub Acute Invasive Aspergillosis
CCPA: Chronic Cavitary Pulmonary Aspergillosis
CFPA: Chronic Fibrosing Pulmonary Aspergillosis
SAFS: Severe Asthma with Fungal Sensitization
ABPA: Allergic Bronchopulmonary Aspergillosis
EUCAST: European Committee on Antibiotic Susceptibility Testing
CLSI: Clinical and Laboratory Standards Institute
RPMI agar: Roswell Park Memorial Institute
MIC: Minimum Inhibitory Concentration
MIC90: Minimum Inhibitory Concentration to inhibit the growth of 90% organisms
ATCC: American Type Culture Collection
